# Artificial intelligence-assisted evaluation of cardiac function by oncology staff in chemotherapy patients

**DOI:** 10.1093/ehjdh/ztae017

**Published:** 2024-02-27

**Authors:** Stella-Lida Papadopoulou, Dimitrios Dionysopoulos, Vaia Mentesidou, Konstantia Loga, Stella Michalopoulou, Chrysanthi Koukoutzeli, Konstantinos Efthimiadis, Vasiliki Kantartzi, Eleni Timotheadou, Ioannis Styliadis, Petros Nihoyannopoulos, Vasileios Sachpekidis

**Affiliations:** Department of Cardiology, Papageorgiou General Hospital, Ring Road, Nea Efkarpia, Thessaloniki 56403, Greece; Department of Medical Oncology, Papageorgiou Hospital, Aristotle University of Thessaloniki, School of Health Sciences, Faculty of Medicine, Ring Road, Nea Efkarpia, Thessaloniki 56403, Greece; Department of Medical Oncology, Papageorgiou Hospital, Aristotle University of Thessaloniki, School of Health Sciences, Faculty of Medicine, Ring Road, Nea Efkarpia, Thessaloniki 56403, Greece; Department of Medical Oncology, Papageorgiou Hospital, Aristotle University of Thessaloniki, School of Health Sciences, Faculty of Medicine, Ring Road, Nea Efkarpia, Thessaloniki 56403, Greece; Department of Medical Oncology, Papageorgiou Hospital, Aristotle University of Thessaloniki, School of Health Sciences, Faculty of Medicine, Ring Road, Nea Efkarpia, Thessaloniki 56403, Greece; Department of Medical Oncology, Papageorgiou Hospital, Aristotle University of Thessaloniki, School of Health Sciences, Faculty of Medicine, Ring Road, Nea Efkarpia, Thessaloniki 56403, Greece; Department of Medical Oncology, Papageorgiou Hospital, Aristotle University of Thessaloniki, School of Health Sciences, Faculty of Medicine, Ring Road, Nea Efkarpia, Thessaloniki 56403, Greece; Department of Cardiology, Papageorgiou General Hospital, Ring Road, Nea Efkarpia, Thessaloniki 56403, Greece; Department of Medical Oncology, Papageorgiou Hospital, Aristotle University of Thessaloniki, School of Health Sciences, Faculty of Medicine, Ring Road, Nea Efkarpia, Thessaloniki 56403, Greece; Department of Cardiology, Papageorgiou General Hospital, Ring Road, Nea Efkarpia, Thessaloniki 56403, Greece; Imperial College London, NHLI Hammersmith Hospital, Du Cane Road, London W120NN, UK; Department of Cardiology, Papageorgiou General Hospital, Ring Road, Nea Efkarpia, Thessaloniki 56403, Greece

**Keywords:** Artificial intelligence, Left ventricular ejection fraction, Point of care, Echocardiography, Cardio-oncology

## Abstract

**Aims:**

Left ventricular ejection fraction (LVEF) calculation by echocardiography is pivotal in evaluating cancer patients’ cardiac function. Artificial intelligence (AI) can facilitate the acquisition of optimal images and automated LVEF (autoEF) calculation. We sought to evaluate the feasibility and accuracy of LVEF calculation by oncology staff using an AI-enabled handheld ultrasound device (HUD).

**Methods and results:**

We studied 115 patients referred for echocardiographic LVEF estimation. All patients were scanned by a cardiologist using standard echocardiography (SE), and biplane Simpson’s LVEF was the reference standard. Hands-on training using the Kosmos HUD was provided to the oncology staff before the study. Each patient was scanned by a cardiologist, a senior oncologist, an oncology resident, and a nurse using the TRIO AI and KOSMOS EF deep learning algorithms to obtain autoEF. The correlation between autoEF and SE–ejection fraction (EF) was excellent for the cardiologist (*r* = 0.90), the junior oncologist (*r* = 0.82), and the nurse (*r* = 0.84), and good for the senior oncologist (*r* = 0.79). The Bland–Altman analysis showed a small underestimation by autoEF compared with SE–EF. Detection of impaired LVEF < 50% was feasible with a sensitivity of 95% and specificity of 94% for the cardiologist; sensitivity of 86% and specificity of 93% for the senior oncologist; sensitivity of 95% and specificity of 91% for the junior oncologist; and sensitivity of 94% and specificity of 87% for the nurse.

**Conclusion:**

Automated LVEF calculation by oncology staff was feasible using AI-enabled HUD in a selected patient population. Detection of LVEF < 50% was possible with good accuracy. These findings show the potential to expedite the clinical workflow of cancer patients and speed up a referral when necessary.

## Graphical Abstract

**Graphical Abstract:**
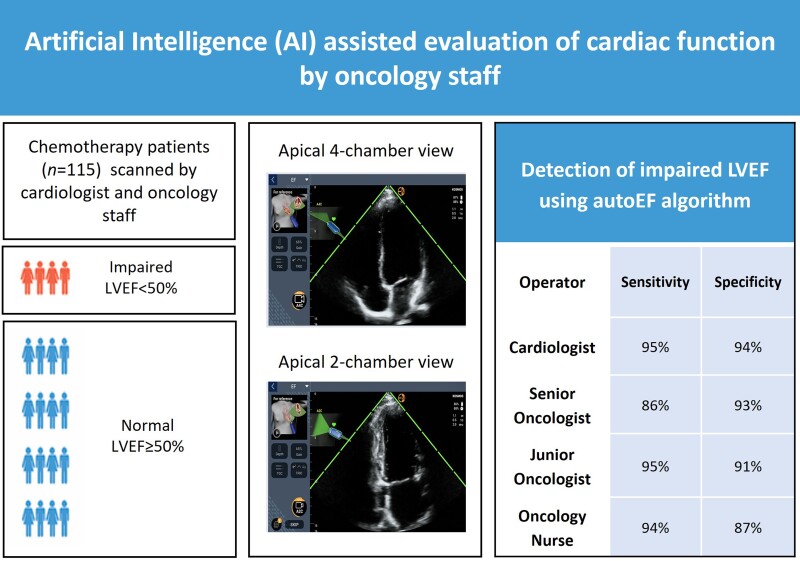
Diagnostic accuracy for impaired LVEF detection by oncology staff in chemotherapy patients using an AI-enabled handheld echocardiography device. AI, artificial intelligence; LVEF, left ventricular ejection fraction.

## Introduction

Population ageing has led to a sustained increase in the incidence of cancer, estimated to reach 23.6 million patients per year globally by 2030.^1^ Advances in early detection and treatment have resulted in a significant improvement in cancer-specific survival.^2^ With prolonged survival, cancer survivors are increasingly subject to late cardiovascular disease related to cardiotoxic cancer therapies compounded by the effect of age-related cardiovascular risk factors.^3^ It is well accepted that most cancer survivors have a higher cardiovascular disease risk than non-cancer controls.^4^

Early recognition of cancer therapy-related cardiac dysfunction (CTRCD) provides an opportunity to mitigate cardiac injury and the risk of developing late cardiac events.^5^ Transthoracic echocardiography is the cornerstone for the detection and surveillance of CTRCD and is the most widely used technique in clinical practice because of its availability, feasibility, and cost-effectiveness.^6^ Although far from perfect, the calculation of left ventricular ejection fraction (LVEF) is the most widely used index of left ventricular (LV) systolic function in clinical practice and is thus pivotal in detecting CTRCD.^7^ Oncologist referrals of patients for the estimation of LVEF pre- and post-chemotherapy have increased during the last decades demanding a significant amount of time and resources from echocardiography laboratories.^8^ Additionally, increased waiting times for service deliverance can lead to treatment delays with potential negative effects on expected outcomes for cancer patients.

Technological advancements over the past two decades have enabled the development of miniaturized handheld ultrasound devices (HUDs) that are compact and battery-operated and can provide echocardiographic images at the point of care with diagnostic image quality. The widespread use of HUDs has the potential to reform the everyday practice of echocardiography, from the exclusive use of the technique by experts in echocardiography laboratories, towards its use by other non-expert users in various settings.^9^ Obviously, adequate training of operators in image acquisition, analysis, interpretation, and reporting is crucial to ensure quality in cardiac imaging. Advances in artificial intelligence (AI) technology have enabled the development of algorithms for the real-time guidance of ultrasound probes to acquire optimal images of the heart and calculate LVEF automatically.

The objectives of this study were to test the ability of the oncology staff as non-expert users to (i) reliably calculate LVEF compared with manually traced biplane Simpson’s rule by a cardiologist on cart-based machines and (ii) accurately identify impaired LV function (LVEF < 50%) in real-time using an AI-enabled HUD in a selected population of oncology patients.

## Methods

### Patient population

Our study group comprised cancer patients examined at the Oncology Day Care Unit of our hospital who were referred to the echocardiography laboratory for the same-day LVEF estimation over an approximate period of 6 months. All patients were >18 years old, haemodynamically stable and were assigned an Eastern Cooperative Oncology Group Performance Status (PS) score of 0 or 1.^10^ Due to the low disease (i.e. impaired LVEF < 50%) prevalence expected in this patient population, a nested case–control diagnostic study design was followed with case:control ratio of 1:4 to ensure adequate power of 80% for the calculation of sensitivity and specificity of the AI-enabled HUD as a screening tool to detect cancer patients with impaired LVEF.^11,12^ Patients with atrial fibrillation or flutter and frequent atrial and ventricular ectopic beats were excluded from the study due to the variation of LVEF between different cardiac cycles, often seen in these populations. Patients with left-sided breast implants, previous extensive thoracic surgeries or structural deformities (i.e. pectus excavatum and pectus carinatum) were also excluded, due to commonly encountered difficulties in acquiring diagnostic images for quantitative estimation of LVEF in such individuals. All patients provided written informed consent and were entered in our single-centre echocardiography database. All treatment decisions were based on standard practice, i.e. LVEF calculation using the biplane Simpson’s rule with standard echocardiography (SE) machines, thus there was no risk to patient safety. The study received proper ethical approval by the institutional scientific board and conformed to the principles of the Declaration of Helsinki.

### Training of oncology staff on the use of artificial intelligence-enabled handheld ultrasound device

**Figure 1:**
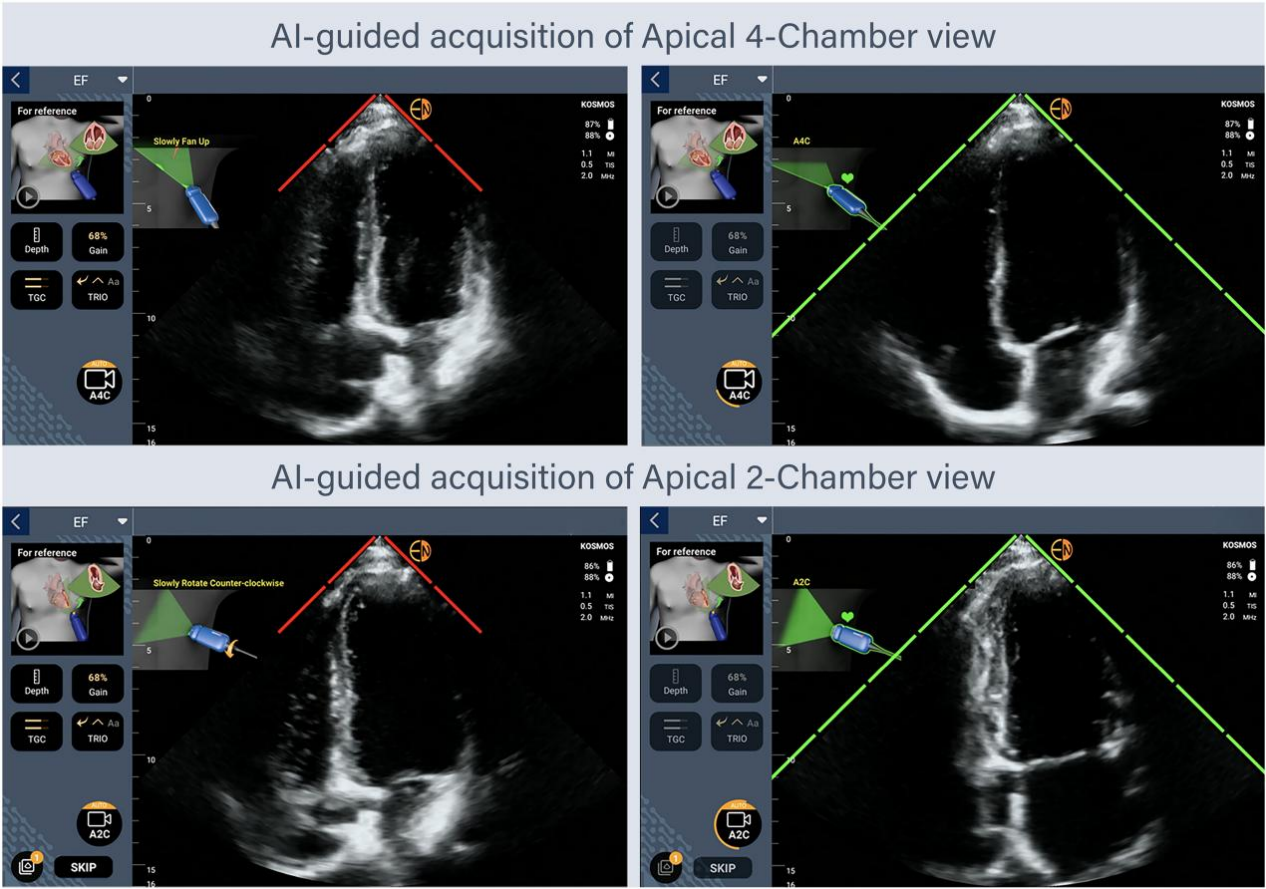
Artificial intelligence-assisted acquisition of optimal apical four-chamber and two-chamber echocardiographic views using the GUIDANCE and GRADING functions of the Kosmos TRIO AI software. A2C, apical two-chamber; A4C, apical four-chamber; AI, artificial intelligence.

In total, five doctors and three nurses working in the oncology department were invited to participate in the study as volunteers. Eventually, five people were recruited: one senior oncologist, two junior oncologists, and three nurses. Based on previously published reports,^13,14^ we concluded that a training programme combining a brief theoretical course and a few hours of hands-on training would be appropriate to enable non-expert users to obtain diagnostic images for the purpose of this project. Before the recruitment of patients, a focused theoretical short course (two sessions of 2-h lectures) on echocardiography fundamentals was provided to the oncology staff participating in the study. Subsequently, the participants attended 2-h sessions of hands-on training using the HUD on human models twice per week for a period of 1 month. The device (Kosmos, EchoNous, Inc.) used for this study is equipped with a 2- to 5-MHz phased-array transducer and uses deep learning algorithms (software version 3.0.1.78) to facilitate image acquisition (TRIO AI algorithm) and calculate LVEF automatically (KOSMOS EF algorithm). In specific, using the functions of the TRIO AI software of the Kosmos HUD under the supervision of a cardiologist, the inexperienced operator conducting the examination was trained to acquire both apical four-chamber (A4C) and apical two-chamber (A2C) views of the heart. The GUIDANCE function provides the operator real-time guidance of the ultrasound probe position to acquire optimal images of the heart by giving simple instructions such as ‘tilt down’, ‘rotate clockwise’, etc. to the user. At the same time, the GRADING function denotes the quality of the live image by displaying a visual five-bar scale next to the image, based on the American College of Emergency Physicians quality assurance grading scale^15^ (*Figure 1*); images with more than three out of five bars are considered to be of sufficient diagnostic quality. After the acquisition of these two views, the device itself automatically estimates LVEF using the KOSMOS EF, a previously clinically validated AI-assisted automated LVEF (autoEF) algorithm.^16^ More detailed description of the algorithms is available as Supplementary material online.

During the training programme, one junior oncologist and two nurses failed to attend the majority of hands-on sessions and dropped-out, due to an increased workload combined with a lack of commitment or interest. Therefore, they did not proceed with the rest of the study. Finally, the remaining oncology staff (a senior oncologist, a junior oncologist, and an oncology nurse) performed successful supervised scanning of five consecutive real-world patients using the HUD to complete the training. A detailed demonstration of the AI-guided image acquisition for LVEF calculation is provided in Supplementary material online, *Video S1*.

### Evaluation of left ventricular ejection fraction by standard echocardiography

The patients included in the study were initially scanned at the echocardiography laboratory. All echocardiographic examinations were performed using a commercially available cart-based system (IE33; Philips Healthcare). Images were acquired by an expert investigator (V.S.—Level 3 training in echocardiography with 15 years of experience), following a standardized protocol. The 2D views used in our study were A4C and A2C views with the patient in the left lateral decubitus position. Images were optimized to improve signal-to-noise ratio and provide optimal endocardial definition. The LV endocardium was used as the boundary for volumetric measurements. Papillary muscles and visible trabeculae were part of the blood pool. If the endocardial border was indistinguishable, non-visible parts were interpolated manually. The image quality for each examination was visually assessed and classified as good, moderate, and poor based on the number of LV walls (septal, anterior, lateral, and inferior) that endocardial borders were not clearly definable in end-diastole (0, 1, or ≥2, respectively). The modified biplane Simpson’s method of discs was used to determine LV volumes and function. End-diastolic and end-systolic endocardial borders were traced manually on frozen 2D images obtained from the A4C and A2C views to derive end-diastolic volume (EDV) and end-systolic volume (ESV). End-diastole was defined at the peak of the electrocardiographic R-wave and/or one frame before mitral valve closure. End-systole was defined as one frame before the mitral valve opening or when ESV was deemed the smallest by the operator. The LVEF was calculated according to the formula ejection fraction (EF) = (EDV−ESV)/EDV × 100%, and all measurements were stored in the digital archive of the echocardiography laboratory as part of the complete echocardiographic study which could be later retrieved. The SE–EF values already calculated were manually entered into our database and were considered the reference values for all comparisons. Furthermore, based on the SE–EF measurements, the LVEF < 50% was considered a clinically relevant cut-off point to define abnormal LV systolic function for oncology patients, since it is a commonly used threshold for deferring chemotherapy.

### Calculation of left ventricular ejection fraction by artificial intelligence-enabled handheld ultrasound device

All the study patients were immediately transferred to the Oncology Day Care Unit and were subsequently scanned with the Kosmos HUD by an independent cardiologist (S.-L.P.), a senior oncologist (D.D.), a junior oncologist (K.L.), and an oncology nurse (S.M.) in a blinded fashion. All the observers were unaware of the LVEF measurement obtained in the echocardiography laboratory. Like the SE, the 2D views acquired were the A4C and A2C views with the patient in the left lateral decubitus position. The TRIO AI software of the device (GUIDANCE and GRADING functions) was used as previously described to facilitate scanning. The maximum allowed scanning time for each operator was 8 min per patient and the acquisition was considered successful only if the images were labelled as diagnostic according to the GRADING (three or more green bars present) and GUIDANCE (optimal view—no suggestions to improve image alignment displayed on the screen) functions for both A4C and A2C views. Fully automated estimation of the LVEF was possible after the acquisition of these two views within 5 s by the KOSMOS EF algorithm at the point of care, and the result was immediately displayed on the screen and saved without any correction by manual tracing. The acquired cardiologist’s Kosmos 2D data (both A4C and A2C views) for each patient was visually assessed for image quality and classified as good, moderate, and poor in a similar way as described above for the SE system images. All acquired autoEF measurements were stored in the handheld device used for the study; they were retrieved after the completion of the recruitment and manually entered to our database.

### Test–retest reliability of artificial intelligence-enabled acquired left ventricular ejection fraction measurements

In 22 randomly selected patients, a second independent AI-enabled acquisition of LVEF was obtained after ∼1 h by a non-expert observer (the senior oncologist), and the test–retest intra-observer reliability of the method was evaluated.

### Statistical analysis

Normal distributions of variables were checked before analysis. Continuous variables were expressed as means ± standard deviation or medians with interquartile range (IQR) when not normally distributed; categorical variables were presented as counts and/or percentages. Agreement between SE–EF and HUD autoEF measurements (primary outcome) was evaluated by Pearson’s correlation coefficient *r* (where *r* = 0 indicates no relationship and +1.0 or −1.0 reflects a strong positive or negative relationship, respectively) and Bland–Altman analysis.^17^ The 95% limits of agreement (LOA) were defined as the range of values between ±1.96 SD from the mean difference. The learning curve for the AI-based EF calculation by the observers was assessed by comparing the correlation coefficient *r* values for the 1st and 2nd half of the patient population (before and after the 50th percentile, respectively). Comparison between continuous variables was performed using paired Student’s *t*-test or analysis of variance with Bonferroni’s correction in post-hoc tests, whereas the variables not normally distributed were compared with the non-parametric Wilcoxon signed-rank test and the Friedman test. Categorical variables were compared using the *χ*^2^ test. Sensitivity, specificity, positive predictive value, negative predictive value, and overall diagnostic accuracy of KOSMOS EF to detect LVEF < 50% (secondary outcomes) were calculated using 2 × 2 contingency tables for each operator as simple measures of the rate of false recommendations by the AI system, and the corresponding 95% confidence intervals (CIs) were determined. Inter-observer reproducibility of the AI-enabled acquisition of LVEF measurements between different operators was calculated by means of the intraclass correlation coefficient (ICC) for average measures with a two-way random model with interaction for the absolute agreement.^18^ Although there is no strict ICC value that marks the cut-off for appropriate correlation, it is commonly accepted that ICC < 0.5 indicates poor correlation, ICC between 0.5 and 0.75 indicates moderate correlation, ICC between 0.75 and 0.9 indicates good correlation, and ICC > 0.90 indicates excellent correlation. Test–retest reliability of the AI-enabled acquisition of EF measurements was assessed using the ICC for single measures (two-way mixed model with interaction for the absolute agreement), linear regression analysis, and the minimal detectable change (MDC) which represents the minimal change required to ascertain that the differences observed reflect a real change rather than measurement error with at least 95% confidence.^19^ The standard error of measurement (SEM) was computed as SD√1−ICC and subsequently the MDC was calculated as 1.96 × √2×SEM. For all statistical tests, a two-tailed *P* value < 0.05 was considered statistically significant. Statistical analysis was performed using SPSS software, version 22.0 (IBM Inc., Chicago, IL, USA).

## Results

### Study population

**Figure 2:**
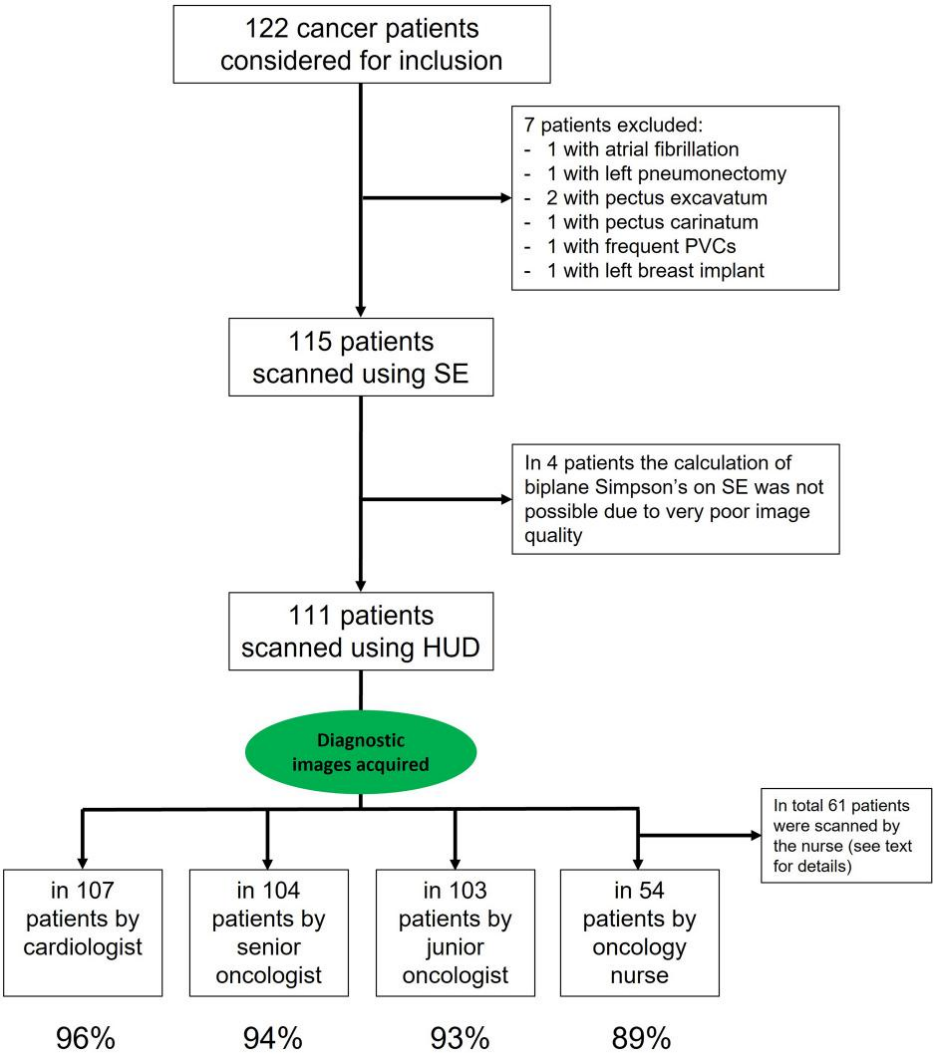
Flowchart of the study population. HUD, handheld ultrasound device; PVC, premature ventricular contraction; SE, standard echocardiography.

In total, 122 cancer patients with PS ≤ 1 who were referred to the echocardiography laboratory for LVEF estimation were considered for inclusion. Among them, seven patients were excluded because they did not fulfil the inclusion criteria (flowchart of the study is presented in *Figure 2*). The study prospectively included 115 patients (mean age 59 ± 12 years, 53% male); 23 patients with impaired LVEF < 50% and 92 patients with normal LVEF. The clinical characteristics of the study population are presented in *Table 1*. The age range of the oncology staff (non-expert users) participating in the study was 32–50 years old.

**Table 1:** Clinical characteristics of the study population (*n* = 115)

Patient characteristics	
Age (years)	59 ± 12
Male gender	53
BMI (kg/m^2^)	25.9 ± 4.6
Heart rate (beats per minute)	74 ± 12
**Baseline CV risk factors**	
HF/cardiomyopathy	20.9
MI or PCI or CABG	13.9
Hypertension	20.9
DM	13.0
Hyperlipidemia	15.6
Obesity (BMI > 30 kg/m^2^)	22.2
Smoking	36.5
**Main oncological diagnosis**	
Gastrointestinal	27.6
Genitourinary	15.2
Breast	14.3
Gynecological	14.3
Head and neck	9.5
Thoracic	9.5
Sarcoma	5.7
Central nervous system	1.9
Other^a^	1.9
**Potentially cardiotoxic chemotherapy**	
Anthracycline chemotherapy	15.7
HER-2 targeted therapies	10.4
VEGF inhibitors	17.4
RAF and MEK inhibitors	0.8
**Chest radiotherapy**	9.6

Values are mean ± SD or percentage (%) of patients unless otherwise indicated.

BMI, body mass index; CABG, coronary artery bypass graft; CV, cardiovascular; DM, diabetes mellitus; HER-2, human epidermal receptor 2; HF, heart failure; LVEF, left ventricular ejection fraction; MEK, mitogen-activated extracellular signal-regulated kinase; MI, myocardial infarction; PCI, percutaneous coronary intervention; RAF, rapidly accelerated fibrosarcoma; VEGF, vascular endothelial growth factor.

^a^Neuroendocrine tumour, skin cancer.

### Feasibility

All the patients were initially scanned with the SE machine and for four of them the calculation of LVEF using biplane Simpson’s rule (reference standard) was not possible due to very poor image quality. The remaining 111 patients were subsequently scanned using the HUD by the independent cardiologist, the senior oncologist, and the junior oncologist. The oncology nurse scanned only 61 patients due to the increased workload during the COVID-19 pandemic. The acquisition of diagnostic images for LVEF calculation using the AI-enabled HUD was feasible in 96% (107/111) of cases for the cardiologist, 94% (104/111) of cases for the senior oncologist, 93% (103/111) of cases for the junior oncologist, and 89% (54/61) of cases for the oncology nurse.

The image quality for the SE system acquisition was visually assessed as good in 30%, moderate in 51%, and poor in 19% of cases and for the HUD acquisition as good in 21%, moderate in 54%, and poor in 25% of cases.

### Method agreement for left ventricular ejection fraction calculation

**Figure 3:**
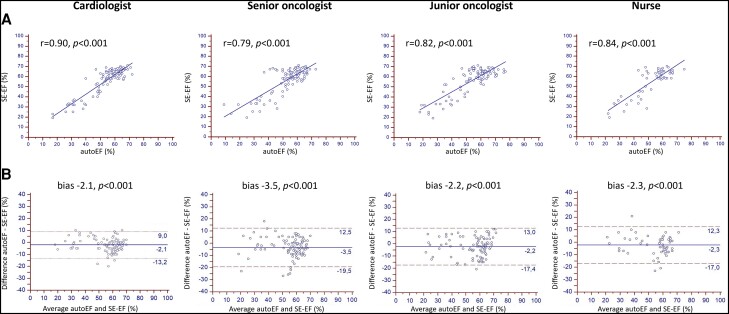
Method agreement for left ventricular ejection fraction calculation. Scatter plots (Panel A) and Bland–Altman plots (Panel B) between the handheld ultrasound device autoEF algorithm and manual biplane Simpson’s ejection fraction measurements on the standard echocardiography system for each operator. EF, ejection fraction; SE, standard echocardiography.

There was a good correlation between the calculated SE–EF and HUD autoEF for all operators (Pearson’s *r* = 0.90 for the cardiologist, *r* = 0.79 for the senior oncologist, *r* = 0.82 for the junior oncologist, and *r* = 0.84 for the nurse, *P* < 0.001 for all), as illustrated in *Figure 3A*. The corresponding Bland–Altman plots in *Figure 3B* showed a small systematic underestimation of LVEF by the HUD autoEF algorithm compared with the SE–EF for all the operators. There was bias −2.1% with LOA 11.1% for the cardiologist, bias −3.5% with LOA 16.0% for the senior oncologist, bias −2.2% with LOA 15.2% for the junior oncologist, and bias −2.3% with LOA 14.6% for the nurse (*P* < 0.001 for all).

### Learning curve for the automated left ventricular ejection fraction calculation

**Figure 4:**
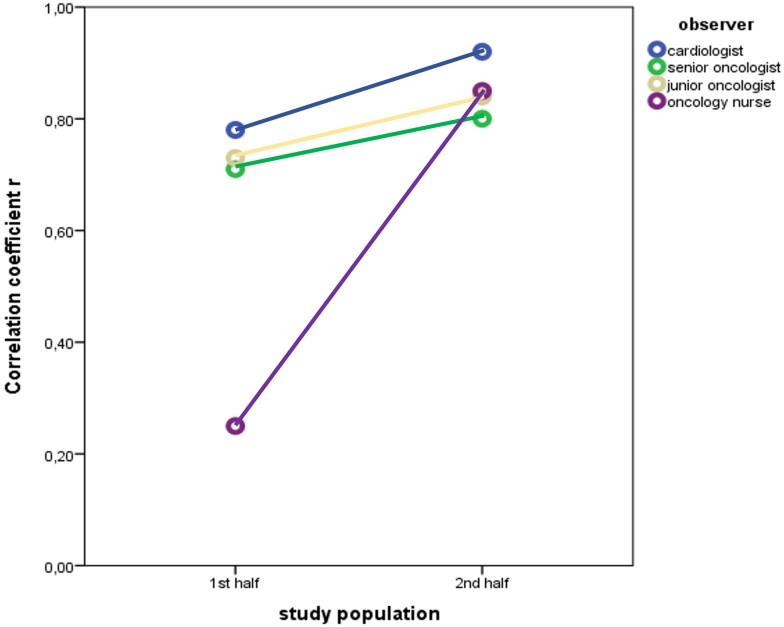
Assessment of the learning curve for the artificial intelligence-based ejection fraction calculation by plotting the correlation coefficient values for the first and second half of the patient population.

The comparison of the correlation coefficient *r* values for the first and second half of the patient population revealed that the diagnostic accuracy of the operators for the AI-based EF calculation improved over time since the correlation between EF measurements was higher (although not reaching statistical significance for all users) for the second half of patient population for all observers (*Table 2* and *Figure 4*).

**Table 2:** Method agreement through the learning curve for the artificial intelligence-based ejection fraction calculation using the handheld ultrasound device

Correlation coefficient *r*				
Operator	For the 1st half of study population	For the 2nd half of study population	For the total study population	*P*-value^a^
**Cardiologist**	0.78	0.92	0.9	0.006
**Senior oncologist**	0.71	0.8	0.79	0.288
**Junior oncologist**	0.73	0.84	0.82	0.142
**Oncology nurse** ^b^	0.25	0.85	0.84	<0.001

^a^Comparison of correlation coefficients for 1st half vs. 2nd half of study population.

^b^Analysis for the oncology nurse was performed using data from 61 patients.

### Diagnostic accuracy to detect impaired left ventricular ejection fraction < 50%

In general, the diagnostic accuracy was good for all the operators, as presented in *Table 3*. Detection of impaired LVEF < 50% by the HUD autoEF algorithm was feasible with a sensitivity of 95% and specificity of 94% for the cardiologist, sensitivity of 86% and specificity of 93% for the senior oncologist, sensitivity of 95% and specificity of 91% for the junior oncologist, and sensitivity of 94% and specificity of 87% for the nurse.

**Table 3:** Diagnostic accuracy for the detection of impaired LVEF using the handheld ultrasound device autoEF algorithm

Operator	Sensitivity (95% CI)	Specificity (95% CI)	PPV (95% CI)	NPV (95% CI)	Overall accuracy
**Cardiologist**	95% (77%–99%)	94% (87%–98%)	81% (64%–91%)	99% (92%–100%)	94%
**Senior oncologist**	86% (65%–97%)	93% (85%–97%)	76% (59%–87%)	96% (90%–99%)	91%
**Junior oncologist**	95% (76%–100%)	91% (83%–96%)	74% (58%–85%)	99% (92%–100%)	92%
**Oncology nurse** ^a^	94% (70%–100%)	87% (73%–95%)	71% (54%–84%)	98% (85%–100%)	89%

NPV, negative predictive value; PPV, positive predictive value.

^a^Diagnostic accuracy for the oncology nurse was calculated using data from 61 patients.

### Inter-observer reproducibility

**Figure 5:**
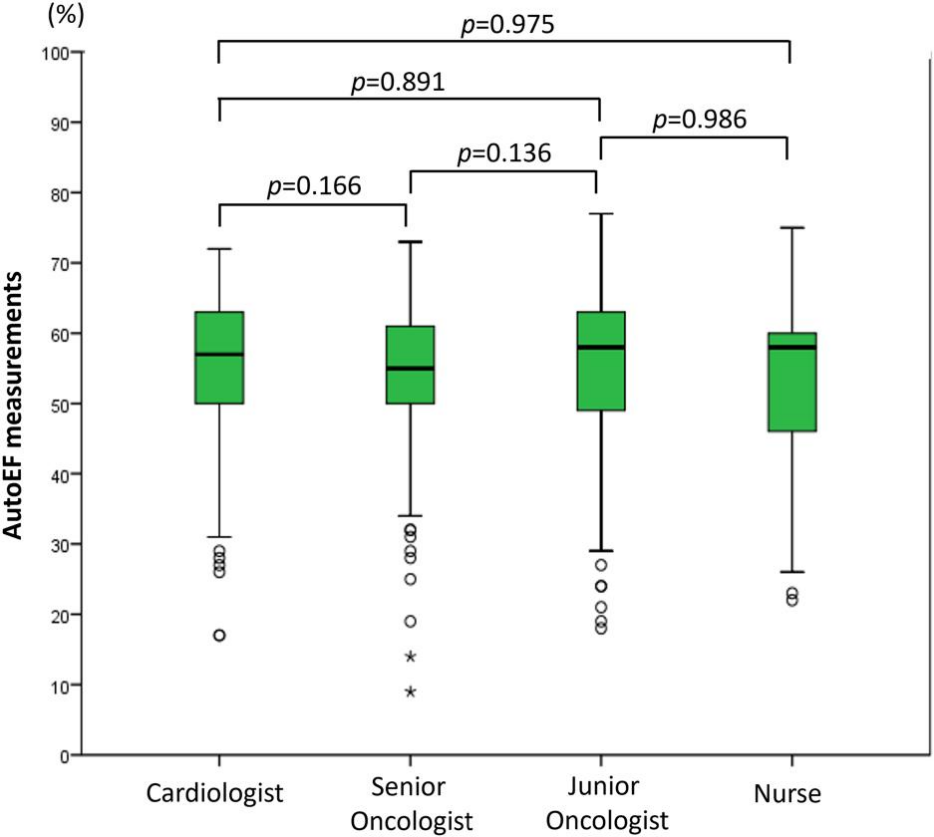
Comparison of automated left ventricular ejection fraction measurements between operators. Box plots of ejection fraction measurements derived by the handheld ultrasound device autoEF algorithm for the different observers. The *P*-values are non-significant for all paired comparisons.

The inter-observer reproducibility for the acquired autoEF measurements was deemed as excellent overall (ICC = 0.96; 95% CI: 0.94–0.97, *P* < 0.001). Finally, there was no statistically significant difference in autoEF measurements between operators for each patient: median autoEF measurement was 57% (IQR 48–62%) for cardiologist, 55% (IQR 50–61%) for senior oncologist, 58% (IQR 46–62%) for junior oncologist, and 57% (IQR 45–61%) for oncology nurse (*P* = 0.708), *Figure 5*.

### Test–retest reliability analyses

**Figure 6:**
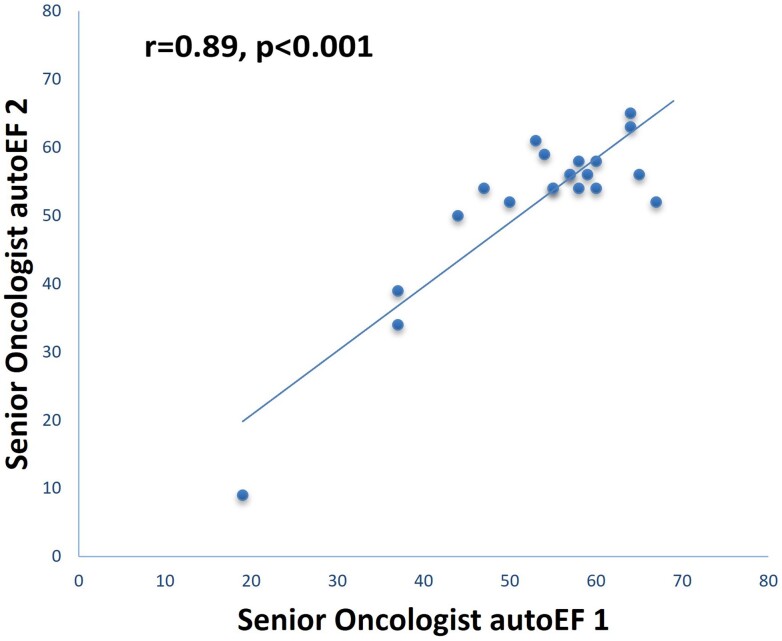
Scatterplot and linear regression between the two repeated artificial intelligence-enabled acquisitions of left ventricular ejection fraction by the senior oncologist.

For 1 of the 22 patients scanned for the test–retest analysis, successful AI-enabled acquisition was not possible by the non-expert operator. In the remaining 21 patients, the intra-observer test–retest reliability for AI-enabled acquisition of LVEF measurements 1 and 2 was deemed as very good (ICC = 0.89; 95% CI: 0.74–0.95, *P* < 0.001). The results of linear regression analysis revealed a correlation coefficient *r* = 0.89, *P* < 0.001 (*Figure 6*). There was no statistically significant difference in autoEF measurements between AI-enabled acquisitions 1 and 2 (mean bias ± SD: −1.2 ± 5.6%, *P* = 0.326). The calculated MDC for the repeated AI-enabled acquisition of LVEF measurements was 5.3%.

## Discussion

To our knowledge, this is the first study to investigate the ability of oncology staff to evaluate LVEF in chemotherapy patients at the point of care. The main finding of this study was that a fully automated measurement of LVEF acquired by oncology staff is feasible by using a novel AI-enabled HUD in a selected population of cancer patients. The acquired measurements are reproducible and comparable to the ones derived by standard biplane Simpson’s method. The AI-assisted autoEF algorithm was able to identify impaired LVEF < 50% with good diagnostic accuracy compared with the cart-based echocardiography systems when used by oncology staff, especially junior doctors.

It is well established that the calculation of LVEF is pivotal for clinical decision-making in cardio-oncology.^20^ Artificial intelligence technology can provide new possibilities to generate accurate, consistent, and automated interpretation of echocardiography exams.^21^ Recently, these AI-assisted algorithms for automated LVEF calculation were made available on HUDs and previous studies have demonstrated a good performance with experienced cardiologists as operators.^16,22^ Since the use of HUDs by non-expert users is already becoming more frequent in different clinical settings, applying AI algorithms that help clinicians acquire optimal images and proceed to accurate measurements may become an important step towards the end of safely expanding cardiac ultrasound to less experienced operators. Indeed, the agreement between experts using SE and non-experts (general practitioners) using a HUD to visually identify patients with reduced LVEF has been reported as poor in an early study.^23^ Moreover, when tested in a population of patients with suspected heart failure, the sole addition of an autoEF algorithm for automatic quantification of LVEF by non-experts (general practitioners and nurses), without ensuring optimal image acquisition, showed modest feasibility, agreement, and reliability compared with experts, precluding implementation into clinical practice.^24^ The development and use of AI algorithms for the real-time guidance of ultrasound probes to acquire optimal images of the heart may play a key role in decreasing the inconsistency of results between experienced and novice users. Early studies showed evidence that a machine-learning algorithm can guide non-experts such as medical students and nurses to acquire diagnostic images and provide an automated LVEF calculation with good agreement with expert users.^14,25^ Similarly, in our investigation, the AI-assisted diagnostic image acquisition by the oncology staff was successful in most patients (feasibility range from 89 to 94%) and the autoEF algorithm allowed detection of LVEF < 50% with high diagnostic accuracy. Further supporting this hypothesis, the use of the TRIO AI algorithm resulted in improved agreement results between SE–EF and autoEF also for the expert (cardiologist), when compared with the results of our previous study in which TRIO AI was not used.^16^

Another important issue is the reproducibility of LVEF measurements in the same patient especially when performed by different operators. A study with patients undergoing chemotherapy for breast cancer reported that 3D echocardiography was the most reproducible technique for LVEF measurement^26^; nevertheless, 3D echocardiography is not widely available and/or used in daily practice. Our results demonstrated that the automated measurements provided by the autoEF algorithm are highly reproducible between the observers. For the serial follow-up of patients, test–retest reliability (also called reproducibility or repeatability) of a technique is also crucial. Test–retest variability describes the variability of separately acquired and interpreted echocardiographic measurements of the same patient. Our results in a small sample of 21 patients of our population demonstrated that the AI-enabled acquired repeated measurements are reliable; in addition, the calculated MDC value of 5.3% is comparable to the test-retest reliability reported in the literature for the biplane Simpson’s method used in SE for LVEF estimation, with MDC ranging from 4.4 to 18.1%.^26–29^ Also, it is similar to the MDC of 4.38% reported in our previous study when the autoEF algorithm was tested by an expert.^16^ Of note, the calculated MDC is below the 10% threshold that is often used in clinical practice to designate a meaningful change in LVEF in several clinical scenarios, such as the follow-up of cancer patients.

Despite the progress in cancer therapies which has led to the improved survival and quality of life,^30^ cardiovascular disease is the leading cause of late morbidity and death among cancer survivors.^31,32^ However, despite the recognized cardiotoxicity of chemotherapeutic drugs, even patients at high risk of cardiovascular complications do not always receive a pre- or post-chemotherapy echocardiogram as indicated. Real-world data from a recent retrospective cohort study showed that pre-chemotherapy echocardiography was obtained in 65% of patients, but only in 19% of them after completing chemotherapy.^33^ Consequently, the feasibility of accurate and reproducible calculation of LVEF by oncology staff at the point of care shown in this study can have significant clinical implications. By using robust guiding and measurement AI algorithms, it could be applied as an initial screening tool pre- and post-chemotherapy for timely detection of cardiac dysfunction when SE examination is not readily available; this strategy could potentially expedite clinical care for cancer patients in case of impaired LVEF detection and speed up referral to a cardio-oncology clinic, where a more thorough cardiac evaluation beyond the mere LVEF measurement can be performed.^34^ It must be emphasized that the role of HUD is not to replace SE but to help identify individuals who must be further investigated by SE. Appropriate training of the operators using HUDs is also mandatory and AI may help towards this end.

### Limitations

The study was performed in a selected cancer patient population with PS of ≤1 and without previous thoracic surgeries, structural deformities, or left-sided breast implants; thus, the results may not be necessarily applicable to all cancer patients. A nested case–control diagnostic study design was used, which could result in a spectrum bias. Patients with very bad image quality in whom the biplane Simpson’s rule could not be applied on the SE system were excluded since no standard method to compare the performance of the autoEF algorithm would be present; nevertheless, this was only 3.5% of our population and most importantly we included cases with poor image quality, contrary to other studies. In this study, we did not perform any global longitudinal strain measurements since it was outside the scope of our study. Finally, given the inclusion of a relatively small number of patients, the possibility of a Type II error should be considered, and this is especially relevant for the diagnostic accuracy results obtained for the nurse because of the lower number of patients scanned.

## Conclusions

A fully automated calculation of LVEF by oncology staff is feasible by using a novel AI-enabled HUD in a selected population of cancer patients. The AI-assisted autoEF algorithm was able to identify LVEF < 50% with good diagnostic accuracy compared with the SE systems. These findings show the potential to expedite the clinical workflow of cancer patients, allowing for early detection of cardiac dysfunction when SE examination is not readily available and speed up a referral to a cardio-oncology clinic when necessary.

## Supplementary Material

ztae017_Supplementary_Data

## Data Availability

The data underlying this article will be shared on reasonable request to the corresponding author.

## References

[1] Thun MJ , DeLancey JO, Center MM, Jemal A, Ward EM. The global burden of cancer: priorities for prevention. Carcinogenesis 2010;31:100–110. 19934210 10.1093/carcin/bgp263 PMC2802672

[2] Miller KD , Nogueira L, Devasia T, Mariotto AB, Yabroff KR, Jemal A, et al Cancer treatment and survivorship statistics, 2022. CA Cancer J Clin 2022;72:409–436. 35736631 10.3322/caac.21731

[3] Abdel-Qadir H , Austin PC, Lee DS, Amir E, Tu JV, Thavendiranathan P, et al A population-based study of cardiovascular mortality following early-stage breast cancer. JAMA Cardiol 2017;2:88–93. 27732702 10.1001/jamacardio.2016.3841

[4] Armenian SH , Xu L, Ky B, Sun C, Farol LT, Pal SK, et al Cardiovascular disease among survivors of adult-onset cancer: a community-based retrospective cohort study. J Clin Oncol 2016;34:1122–1130. 26834065 10.1200/JCO.2015.64.0409 PMC7357493

[5] Cardinale D , Colombo A, Bacchiani G, Tedeschi I, Meroni CA, Veglia F, et al Early detection of anthracycline cardiotoxicity and improvement with heart failure therapy. Circulation 2015;131:1981–1988. 25948538 10.1161/CIRCULATIONAHA.114.013777

[6] Lyon AR , Lopez-Fernandez T, Couch LS, Asteggiano R, Aznar MC, Bergler-Klein J, et al 2022 ESC guidelines on cardio-oncology developed in collaboration with the European Hematology Association (EHA), the European Society for Therapeutic Radiology and Oncology (ESTRO) and the International Cardio-Oncology Society (IC-OS). Eur Heart J 2022;43:4229–4361. 36017568 10.1093/eurheartj/ehac244

[7] Liu J , Banchs J, Mousavi N, Plana JC, Scherrer-Crosbie M, Thavendiranathan P, et al Contemporary role of echocardiography for clinical decision making in patients during and after cancer therapy. JACC Cardiovasc Imaging 2018;11:1122–1131. 30092969 10.1016/j.jcmg.2018.03.025

[8] Rahimi AR , York M, Gheewala N, Markson L, Hauser TH, Manning WJ. Trends in outpatient transthoracic echocardiography: impact of appropriateness criteria publication. Am J Med 2011;124:740–746. 21787903 10.1016/j.amjmed.2011.03.030

[9] Cardim N , Dalen H, Voigt JU, Ionescu A, Price S, Neskovic AN, et al The use of handheld ultrasound devices: a position statement of the European Association of Cardiovascular Imaging (2018 update). Eur Heart J Cardiovasc Imaging 2019;20:245–252. 30351358 10.1093/ehjci/jey145

[10] West HJ , Jin JO. JAMA oncology patient page. Performance status in patients with cancer. JAMA Oncol 2015;1:998. 26335750 10.1001/jamaoncol.2015.3113

[11] Bujang MA , Adnan TH. Requirements for minimum sample size for sensitivity and specificity analysis. J Clin Diagn Res 2016;10:YE01–YYE6. 10.7860/JCDR/2016/18129.8744 PMC5121784 27891446

[12] Biesheuvel CJ , Vergouwe Y, Oudega R, Hoes AW, Grobbee DE, Moons KG. Advantages of the nested case-control design in diagnostic research. BMC Med Res Methodol 2008;8:48. 18644127 10.1186/1471-2288-8-48 PMC2500041

[13] Cawthorn TR , Nickel C, O'Reilly M, Kafka H, Tam JW, Jackson LC, et al Development and evaluation of methodologies for teaching focused cardiac ultrasound skills to medical students. J Am Soc Echocardiogr 2014;27:302–309. 24433979 10.1016/j.echo.2013.12.006

[14] Narang A , Bae R, Hong H, Thomas Y, Surette S, Cadieu C, et al Utility of a deep-learning algorithm to guide novices to acquire echocardiograms for limited diagnostic use. JAMA Cardiol 2021;6:624–632. 33599681 10.1001/jamacardio.2021.0185 PMC8204203

[15] American College of Emergency Physicians (ACEP). Emergency ultrasound standard reporting guidelines; 2018. https://www.acep.org/globalassets/uploads/uploaded-files/acep/clinical-and-practice-management/policy-statements/information-papers/emergency-ultrasound-standard-reporting-guidelines---2018.pdf (18 January 2023).

[16] Papadopoulou S-L , Sachpekidis V, Kantartzi V, Styliadis I, Nihoyannopoulos P. Clinical validation of an artificial intelligence-assisted algorithm for automated quantification of left ventricular ejection fraction in real time by a novel handheld ultrasound device. Eur Heart J Digit Health 2022;3:29–37. 36713988 10.1093/ehjdh/ztac001 PMC9707920

[17] Bland JM , Altman DG. Statistical methods for assessing agreement between two methods of clinical measurement. Lancet 1986;327:307–310. 2868172

[18] Koo TK , Li MY. A guideline of selecting and reporting intraclass correlation coefficients for reliability research. J Chiropr Med 2016;15:155–163. 27330520 10.1016/j.jcm.2016.02.012 PMC4913118

[19] Bunting KV , Steeds RP, Slater LT, Rogers JK, Gkoutos GV, Kotecha D. A practical guide to assess the reproducibility of echocardiographic measurements. J Am Soc Echocardiogr 2019;32:1505–1515. 31653530 10.1016/j.echo.2019.08.015

[20] Alexandre J , Cautela J, Ederhy S, Damaj GL, Salem JE, Barlesi F, et al Cardiovascular toxicity related to cancer treatment: a pragmatic approach to the American and European cardio-oncology guidelines. J Am Heart Assoc 2020;9:e018403. 32893704 10.1161/JAHA.120.018403 PMC7727003

[21] Alsharqi M , Woodward WJ, Mumith JA, Markham DC, Upton R, Leeson P. Artificial intelligence and echocardiography. Echo Res Pract 2018;5:R115–RR25. 30400053 10.1530/ERP-18-0056 PMC6280250

[22] Filipiak-Strzecka D , Kasprzak JD, Wejner-Mik P, Szymczyk E, Wdowiak-Okrojek K, Lipiec P. Artificial intelligence-powered measurement of left ventricular ejection fraction using a handheld ultrasound device. Ultrasound Med Biol 2021;47:1120–1125. 33451814 10.1016/j.ultrasmedbio.2020.12.003

[23] Nilsson G , Soderstrom L, Alverlind K, Samuelsson E, Mooe T. Hand-held cardiac ultrasound examinations performed in primary care patients by nonexperts to identify reduced ejection fraction. BMC Med Educ 2019;19:282. 31345207 10.1186/s12909-019-1713-9 PMC6659293

[24] Hjorth-Hansen AK , Magelssen MI, Andersen GN, Graven T, Kleinau JO, Landstad B, et al Real-time automatic quantification of left ventricular function by hand-held ultrasound devices in patients with suspected heart failure: a feasibility study of a diagnostic test with data from general practitioners, nurses and cardiologists. BMJ Open 2022;12:e063793. 10.1136/bmjopen-2022-063793 PMC9562287 36229153

[25] Schneider M , Bartko P, Geller W, Dannenberg V, Konig A, Binder C, et al A machine learning algorithm supports ultrasound-naive novices in the acquisition of diagnostic echocardiography loops and provides accurate estimation of LVEF. Int J Cardiovasc Imaging 2021;37:577–586. 33029699 10.1007/s10554-020-02046-6 PMC7541096

[26] Thavendiranathan P , Grant AD, Negishi T, Plana JC, Popovic ZB, Marwick TH. Reproducibility of echocardiographic techniques for sequential assessment of left ventricular ejection fraction and volumes: application to patients undergoing cancer chemotherapy. J Am Coll Cardiol 2013;61:77–84. 23199515 10.1016/j.jacc.2012.09.035

[27] Baron T , Berglund L, Hedin EM, Flachskampf FA. Test-retest reliability of new and conventional echocardiographic parameters of left ventricular systolic function. Clin Res Cardiol 2019;108:355–365. 30368567 10.1007/s00392-018-1363-7 PMC6426804

[28] Houard L , Militaru S, Tanaka K, Pasquet A, Vancraeynest D, Vanoverschelde JL, et al Test-retest reliability of left and right ventricular systolic function by new and conventional echocardiographic and cardiac magnetic resonance parameters. Eur Heart J Cardiovasc Imaging 2021;22:1157–1167 32793957 10.1093/ehjci/jeaa206

[29] Otterstad JE , Froeland G, St John Sutton M, Holme I. Accuracy and reproducibility of biplane two-dimensional echocardiographic measurements of left ventricular dimensions and function. Eur Heart J 1997;18:507–513. 9076390 10.1093/oxfordjournals.eurheartj.a015273

[30] Siegel RL , Miller KD, Fuchs HE, Jemal A. Cancer statistics, 2022. CA Cancer J Clin 2022;72:7–33. 35020204 10.3322/caac.21708

[31] Daher IN , Daigle TR, Bhatia N, Durand JB. The prevention of cardiovascular disease in cancer survivors. Tex Heart Inst J 2012;39:190–198. 22740730 PMC3384047

[32] Ruddy KJ , Patel SR, Higgins AS, Armenian SH, Herrmann J. Cardiovascular health during and after cancer therapy. Cancers (Basel) 2020;12:3737. 33322622 10.3390/cancers12123737 PMC7763346

[33] Regino CA , Cardona-Vélez J, Simanca JDB, Miranda-Arboleda AF, Arroyave JGG, Jaimes F. Cardio-oncology clinical assessment and screening in patients undergoing high toxicity chemotherapy: a retrospective cohort study. Cureus 2022;14:e32513. 36654601 10.7759/cureus.32513 PMC9838591

[34] Madan N , Lucas J, Akhter N, Collier P, Cheng F, Guha A, et al Artificial intelligence and imaging: opportunities in cardio-oncology. Am Heart J Plus 2022;15:100126. 35693323 10.1016/j.ahjo.2022.100126 PMC9187287

