# Supplementary material for: Artificial intelligence-assisted evaluation of cardiac function by oncology staff in chemotherapy patients
Source: Eur Heart J Digit Health. 2024 Feb 27;5(3):278–87. doi: 10.1093/ehjdh/ztae017 (PMC11104473; doi:10.1093/ehjdh/ztae017)
Supplement: ztae017_Supplementary_Data [file ztae017_supplementary_data.zip › TRIO AI and KOSMOS EF algorithms.docx]

# ALGORITHM DEVELOPMENT

## KOSMOS Device description

KOSMOS is a portable diagnostic medical ultrasound system that has obtained 510K clearance. It is equipped with 128 channels and is specifically designed for clinical assessment of the heart, lungs, and abdomen. The system incorporates an intuitive machine learning (ML) based workflow, making it user-friendly for both experienced and novice healthcare professionals. Its primary purpose is to aid in acquiring essential cardiac views and accurately quantifying left ventricular function in individuals with normal heart function as well as patients in diverse clinical settings.

## Training data for model training

Most of the dataset used in this study was obtained through random sampling from a large clinical database, which encompasses a wide range of patient age and sex, mirroring typical clinical settings. The dataset comprises echocardiography exams acquired from both the Phillips iE33 and CX50 systems, as well as the KOSMOS device. It includes patients with diverse cardiac pathologies, offering a comprehensive representation of various conditions.

The training dataset consists of over one thousand echocardiography exams, which were partitioned into train and test sets using stratified random sampling. Multiple frames were sampled on average per clip to capture comprehensive information. All frames were carefully annotated by experts and extracted for model training. The training data encompasses subjects with a range of cardiac pathologies, including abnormal heart rhythms, apical hypokinesis, congestive heart failure, dilated cardiomyopathy, myocardial infarction, hypertrophic cardiomyopathy, left ventricular hypertrophy, pericarditis/pericardial effusion, pulmonary hypertension, sigmoid septum, aortic stenosis, mitral valve insufficiency, mitral valve prolapse, and pericarditis.

**Training data Demographics**

| **GENDER** | **FEMALE** | **MALE** |  |
| --- | --- | --- | --- |
|  | 41% | 59% |  |
| **PATHOLOGY** | **NORMAL** | **ABNORRMAL** |  |
|  | 54% | 46% |  |
| **AGE** | **18-35 (years)** | **36-55 (years)** | **56 and older (years)** |
|  | 13% | 25% | 62% |
| **BMI** | **Healthy Weight (BMI < 25)** | **Over-weight (25 <= BMI < 30)** | **Obesity (BMI >=30)** |
|  | 39% | 32% | 29% |

## Algorithms Description

## TRIO Software

The Kosmos Trio AI software has three functionalities:

1. AutoLabel: provides real-time automatic labeling of anatomical structures in various ultrasound views, including standard apical 4-chamber (A4C), apical 2-chamber (A2C), apical 3-chamber (A3C), apical 5-chamber (A5C), subcostal 4 chamber (SUBCOSTAL-4C), subcostal inferior vena cava (SUBCOSTAL-IVC), parasternal long-axis (PLAX), right ventricular inflow tract (RVIT), right ventricular outflow tract (RVOT), suprasternal, and parasternal short-axis (PSAX) views at the aortic valve level (PSAX-AV), the mitral valve level (PSAX-MV), the papillary muscle level (PSAX-PM), and the left ventricle apex level (PSAX-AP). The AutoLabel function is designed to assist both qualified and trained medical professionals, as well as those without specialized training in sonography, in obtaining accurate diagnostic quality A4C and A2C views for the measurement of Simpson's Biplane Left Ventricular Ejection Fraction (LVEF) and assessment of left ventricular systolic function.
2. Guidance AI: provides real-time suggestions for adjusting the position of the ultrasound transducer to achieve high-quality A4C and A2C cardiac views. The Guidance function assists users in obtaining optimal diagnostic images by providing real-time feedback on the required adjustments to improve transducer positioning.
3. Grading AI: provides real-time assessment of the image quality of A4C and A2C views. It utilizes the ACEP 5-level quality grading scale, with grades 1-2 representing non-diagnostic images and grades 3-5 representing diagnostic images. The software displays these grading levels as colored bars attached to both sides of the ultrasound cone, allowing users to easily evaluate the image quality.

To train the underlying algorithm, the real-time probe manipulation task is approached as a suboptimal cardiac view identification problem. A team of clinicians and novice ultrasound users defined a set of commonly produced suboptimal cardiac views that negatively influence the diagnostic quality of the ultrasound image due to incorrect probe orientation. The algorithm predicts these suboptimal views, prompting the software to provide text or graphical feedback to guide the user in improving the orientation of the ultrasound image.

The Guidance algorithm takes an ultrasound image frame with a pixel dimension of 128x128 as input. It undergoes processing through a network that includes a Batch Normalization (BN) layer and 9 convolutional blocks (CB). Each CB utilizes a depth-wise separable convolutional design, as described in the paper "MobileNetV2: Inverted Residuals and Linear Bottlenecks" (https://arxiv.org/pdf/1801.04381.pdf), to reduce the number of architecture parameters and enhance model processing speed during inference. The intermediate feature maps are then pooled using a Max-Pool layer and split into two outputs: suboptimal view classification and image quality classification.

For automatic labeling, the software employs a custom CNN architecture. The model incorporates inverse residual linear bottleneck layers based on the principles introduced by MobilenetV2 (https://arxiv.org/pdf/1801.04381.pdf). The final object detection layer is designed to regress for the bounding box coordinates encompassing the anatomical structures of interest using the YOLOv3 framework (https://arxiv.org/abs/1804.02767). The network weights are optimized using AdamW (https://arxiv.org/abs/1711.05101) for 1000 epochs, with learning rate scheduler (https://arxiv.org/abs/1708.07120). The loss function employed for object detection follows the framework described in the YOLOv3 paper (https://arxiv.org/abs/1804.02767).

## KOSMOS EF

KOSMOS EF is an AI-assisted workflow designed for healthcare professionals to accurately calculate the Simpson's Biplane ejection fraction (EF) of the left ventricle (LV). The workflow adheres to the modified Simpson's Biplane LVEF as recommended by the American Society of Echocardiography. The process begins with acquiring image sequences (clips) of the heart in the apical 4-chamber (A4C) and apical 2-chamber (A2C) views. These clips are then processed using a machine learning (ML) algorithm to identify the end-diastolic (ED) and end-systolic (ES) frames in both A4C and A2C clips. Subsequently, the left ventricle (LV) is segmented in all four frames (A4C ED, A4C ES, A2C ED, A2C ES), resulting in four initial contours of the LV.

The computation of the Simpson's based ejection fraction involves the following three steps:

- Detecting the ED and ES frames in the A4C and A2C images.
- Contouring the detected ED and ES frames in the A4C and A2C images.
- Utilizing the method of disks on the A4C and A2C contours to compute the end-diastolic volume (EDV) and end-systolic volume (ESV) for both A4C and A2C clips. The LVEF is then calculated as (LVEDV - LVESV) * 100 / LVEDV. The method of disks estimates a 3D volume from a closed 2D contour by separating the contour into a stacked set of disks.

The ED and ES phases are identified from the A4C and A2C clips using a custom convolutional neural network (CNN) architecture optimized to run efficiently on the proprietary Kosmos platform and off-the-shelf tablets. The neural network consists of cascaded convolutional blocks followed by a max-pooling operation and fully connected layers. It is trained using a root mean square loss function.

To trace the LV endocardial border in the detected ED and ES frames of the A4C and A2C images, a custom and computationally efficient CNN based on the U-Net architecture was developed. The network comprises six convolutional blocks interspersed with MaxPool layers for down-sampling. The extracted image features are then up-sampled using six additional convolutional blocks. Two skip connections are utilized to exchange relevant information between the up-sample and down-sample branches. Finally, the resulting feature map is fed into a SoftMax layer to generate the raw segmentation masks. The LV segmentation model is trained using the dice similarity coefficient loss function. The final post-processed binary LV mask is fed into the method of disks pipeline for volume and EF computation.
